# The supplementation with fermented pine needles (*Pinus ponderosa*) improve performance and jejunal barrier function in weaned pigs

**DOI:** 10.3389/fvets.2026.1914689

**Published:** 2026-08-24

**Authors:** Pei Mao, Wenfeng Ma, Xiaoli Zhang, Zhiqian Xu, Fengyun Wen, Qunhui Liu, Chenggang Yan, Qiujue Wu

**Affiliations:** 1 College of Animal Science and Technology, Henan University of Science and Technology, Luoyang, Henan, China; 2 Agricultural Technology Promotion Center of Chongren County, Fuzhou, Jiangxi, China; 3 Henan Beimuda Agriculture and Animal Husbandry Co., LTD., Xuchang, China

**Keywords:** fermented pine needles, growth performance, immune response, intestinal barrier function, weaned piglets

## Abstract

This study aimed to evaluate the impacts of fermented pine needles on growth performance, intestinal barrier function and immunological reaction of piglets after weaning. In total, 200 28-day-old weaned piglets were selected and randomly allocated into 5 feeding groups: control group (standard basal diet, CON), non-fermented pine needles group (2.0% non-fermented pine needles, NF); and three fermented pine needle groups (FR1, FR2, FR3) supplemented with 1.0%, 2.0% and 3.0% fermented pine needles for 28 d. The data showed that fermentation pine needles supplementation significantly promote average daily gain (ADG), optimize feed conversion ratio (F/G), and decrease the diarrhea morbidity of post-weaning piglets (*P* < 0.05). Compared with the CON and NF groups, serum D-lactic acid diamine oxidase (DAO) activity were significantly reduced in fermented treatment groups (*P* < 0.05). Moreover, on day 14 of the experiment, the serum and jejunal mucosal concentrations of total nitric oxide synthase (TNOS), tumor necrosis factor (TNF)-α, interleukin (IL)-1β, IL-2, and IL-6 were significantly lower in fermented groups than those in the CON group, except for jejunal mucosal IL-1β (*P* < 0.05). Compared with piglets in the control group, dietary fermented pine needles supplementation significantly decreased serum IL-1β and jejunal mucosal TNF-α levels in weaned piglets on day 28 of the experiment (*P* < 0.05). Fermented pine needle supplementation significantly elevated serum and jejunal mucosal *IL-10* contents, as well as the mRNA abundances of *Occludin, ZO-1, Claudin-1*, and *IL-10* in jejunal mucosa compared with the CON group (*P* < 0.05). Meanwhile, fermented pine needle treatments significantly reduced the jejunal mucosal mRNA expression of *nuclear factor kappa-B* (*NF-*κ*B*) and *IL-6* compared to the CON group (*P* < 0.05). In summary, dietary fermented pine needles improve growth performance and reduce diarrhea incidence of weaned piglets, which ia attributed to regulated nitric oxide synthase activity, maintained jejunal barrier integrity, and balanced secretion and gene expression of intestinal cytokines.

## Introduction

Weaning constitutes a critical stress period in piglets, leaving animals susceptible to pathogenic microbes as well as dietary and environmental antigens, which usually causes reduced growth performance, intestinal inflammation and higher diarrhea rate (1). McCracken et al. (2) reported that weaning stress suppresses growth performance and feed intake, and potentially triggers intestinal inflammatory injury and damages villus-crypt morphological structure. Pluske et al. (3) indicated that these intestinal lesions impair small intestinal absorptive function and further reduce feed utilization efficiency. Numerous recent studies have confirmed that weaning stress disrupts the intestinal barrier of piglets, destroys tight junction proteins of intestinal epithelium and increase intestinal permeability (4–6). Moreover, previous investigations have also verified that weaning stress induces abnormal intestinal immune response. Excessive secretion of pro-inflammatory cytokines such as TNF-α, IL-1β, IL-6, and IL-8 subsequently aggravates intestinal mucosal injury and physiological dysfunction (7). Collectively, existing literature fully demonstrates that weaning impairs intestinal mucosal barrier, elevates intestinal permeability, triggers mucosal inflammation, and ultimately leads to poor growth performance, frequent diarrhea and increased mortality. Therefore, exploring efficient green feed additives to alleviate weaning stress damage if of great production value.

Pine needles are a natural herbal feed resource containing citric acid, shikimic acid, terpenoids, phenolic compounds, flavonoids, alkaloids, and monosaccharide (glucose, fructose, galactose, and sucrose), which possess antibacterial, antioxidant and immunomodulatory biological activities (8–11). Published studies have proven supplementation increases small intestinal length and protein digestibility, promotes growth and tissue development of broilers (12), elevates serum antioxidant enzymes levels of laying hens (13), and increases the proportion of CD3~+ CD4~+ T lymphocytes in Roman cocks (10). These results suggest pine needles can serve as functional feed additives to improveantioxidant capacity and relieve weaning stress, with great potential to replace traditional growth-promoting antibiotics. However, raw pine needles contain anti-nutritional factors including tannins, phenols and phytates; microbial fermentation can effectively degrade these anti-nutrients, reduce malondialdehyde accumulation and improve total superoxide dismutase activity in broilers (14, 15). To date, systematic research regarding the regulatory effects of fermented pine needles on weaned piglets, especially intestinal barrier and immune function, remains insufficient. Thus, the present study was designed to assess the impacts of fermented pine needles supplementation on growth performance, gut mucosal barrier function and immune reaction in weaning pigs.

## Materials and methods

### Processing and preparation of microbially fermented pine needles materials

Fresh pine needle sampling, *Aspergillus niger*, strain activation and cultivation and fermented pine needle materials preparation were conducted strictly following the protocol of Wu et al. (15). The proximate nutrient composition and bioactive component detection data of unfermented and fermented *Pinus ponderosa* needles have been fully reported in our previously published independent research (15).

### Animal management, dietary formulation, and experimental design

In the present 28-d feeding trial, 200 healthy crossbred weaned piglets (Duroc × Landrace × Yorkshire, initial body weight (6.26 ± 0.11 kg, *n* = 200) with an age of 28 days were selected as experimental animals. Piglets were randomly divided into five groups, with five replicates per treatment and eight piglets per replicate. Experimental treatments were set as follows: CON group (basal diet without pine needles), NF group (basal diet supplemented with 2.0% unfermented pine needles), FR1 group (basal diet + 1.0% fermented pine needles), FR2 group (basal diet + 2.0% fermented pine needles), and FR3 group (basal diet + 3.0% fermented pine needles). All piglets were raised in individual stainless steel wire cages (120 cm × 80 cm), each equipped with nipple drinkers and feeding troughs to ensure free access to water and feed. The corn-soybean basal diet was formulated in accordance with the swine nutritional standards recommended by National Research Council of the National Academies (16), and. detailed dietary composition is displayed in Table 1. Fermented and unfermented pine needle raw materials were added extraneously on a mass basis. All animal experimental operations and feeding management schemes were reviewed and approved by the Animal Care and Use Committee of Henan University of Science and Technology.

**Table 1:** Composition and nutrient levels of the basal diet (air-dry basis, %).

Ingredients	Content (%)	Nutrient level	Content (%)
Corn	59.57	Calculated chemical composition	
Soybean meal	13.00	Digestible energy (MJ/kg)	14.39
Wheat bran	4.00	Crude protein	18.92
Extruded soybean	9.50	Ca	0.83
Concentrated soy protein	3.10	Total phosphorus	0.64
Whey powder	3.90	Available phosphorus	0.41
Soybean oil	1.00	D-Lysine	1.46
Sucrose	1.50	D-Methionine	0.47
CaCO_3_	0.70	D-Met+D-Cys	0.65
CaHPO_4_	0.70	D-Threonine	0.83
NaHCO_3_	0.50	D-Tryptophan	0.26
L-Lysine-HCl	0.75		
DL-Methionine	0.22		
L-Threonine	0.18		
L-Tryptophan	0.15		
L-Valine	0.23		
NaCl	0.50		
Vitamin-mineral premix^a^	0.50		
Total	100.00		

^a^Premix provided the following per kg of diets: Vitamin A 2700 IU; Vitamin D_3_ 300 IU; Vitamin E 25 IU; Vitamin K_3_ 0.5 mg; Vitamin B_1_ 1.50 mg; Vitamin B_2_ 5.25 mg; Vitamin B_12_ 0.026 mg; Folic acid, 0.3 mg; Nicotinic acid 22 mg; Pantothenic acid 14 mg; biotin 0.05 mg; Cu (CuSO_4_·5H_2_O) 5.6 mg; Fe (FeSO_4_·H_2_O) 90 mg; Mn 5.2 mg; Zn (ZnSO_4_·H_2_O) 106 mg; Se (Na_2_SeO_3_) 0.3 mg; I (KI) 0.2 mg.

### Growth performance and sample collection

During the pre-experiment period, the breeding facilities, rearing pens, feeding utensils and drinking equipment were fully cleaned and disinfected. The pig house indoor temperature was stabilized at 25–28 °C, with relative humidity controlled at 65%-70%. All piglets were allowed free access to feed and drinking water throughout the trial. Individual piglet body weight and feed consumption of each replicate were recorded on days 0, 14, and 28 of the trial to calculate ADG, ADFI, and F/G ratio.

Diarrhea occurrence was recorded daily during the whole experiment. Diarrhea judgment criteria criteria referred to Cheng et al. (17): piglets with watery feces lasting for two consecutive days were defined as diarrheal individuals. Diarrhea rate was calculated as cumulative number of diarrheal piglets/total experimental pig quantity × whole trial duration. On days 14 and 28 of the trial, five piglets were randomly selected from each treatment for serum and tissue sampling, then humanely euthanized. Blood samples were collected from anterior vena cava, stood for 30 min, centrifuged at 3,500 × g, 4 °C for 15 min, and serum supernatant was separated and stored at −20 °C for subsequent detection. After laparotomy, jejunal segment were rapidly dissected, and 2 cm intestinal segments were intercepted. Jejunal mucosal scrapings were gently collected on ice-cooled sterile slides. All mucosal samples were transferred to cryovials, snap-frozen in liquid nitrogen and preserved at −80 °C for subsequent enzyme activities, cytokine levels and gene expression detection.

### Enzyme activity detection and biochemical analysis

Commercial ELISA kits manufactured by mlbio Co., Ltd. (Shanghai, China) were adopted to determine seru, DAO (pg/mL) and D-lactic acid (μg/mL) concentrations, as well as TNF-α, IL-1β, IL-2, IL-6, and IL-10 levels in serum and jejunal mucosa were also examined.

Homogenates of jejunal mucosa were centrifuged at 3,000 × g, 4 °C for 10 min, and supernatant was collected for spectrophotometric determination of iNOS, TNOS, and NO using matched commercial biochemical kits (Nanjing, China). All operations strictly followed kit manufacturer instructions.

### Real-time quantitative PCR

Intestinal mucosa total RNA was extracted, and RNA purity and concentration were detected via agarose gel electrophoresis and NanoDrop 2000 spectrophotometer. RNA concentration was adjusted to 1 μg/μL for reverse transcribe into cDNA. Real-time fluorescent quantitative PCR was used to detect target gene mRNA expression levels, and detailed reaction systems and procedures are listed in Tables 2, 3 Target gene primers were designed based on NCBI gene sequences, and primer specificity was verified; full primer information is shown in Table 4.

**Table 2:** Primer sequence information.

Gene^a^	Primer sequence (5‘-3‘)	Length (bp)	Accession no.
*GAPDH*	F:GAAGGTCGGAGTGAACGGAT	149	AF017079
	R:CATGGGTAGAATCATACTGGAACA		
*ZO-1*	F:CCAACCATGTCTTGAAGCAGC	215	XM_021098896.1
	R:TGCAGGAGTGTGGTCTTCAC		
*Claudin-1*	F:CTCTCCCCACATTCGAGATGATT	247	NM_001244539.1
	R:AGCCATTGACGTGATCCAGG		
*Occludin*	F:TCAGGTGCACCCTCCAGATT	169	NM_001163647.2
	R:TATGTCGTTGCTGGGTGCAT		
*TNF-α*	F:GCCCTTCCACCAACGTTTTC	128	XM_003126626.6
	R:CAAGGGCTCTTGATGGCAGA		
*NF-κB*	F:AGCCATTGACGTGATCCAGG	248	NM_001048232.1
	R:CGAAATCGTGGGGCACTTTG		
*IL-1β*	F:ACACACCTCTGACTCAAGCC	188	NM_001123042.1
	R:GGGGCCATCAGCCTCAAATA		
*IL-6*	F:TTCAGTCCAGTCGCCTTCT	91	NM_214399.1
	R:GTGGCATCACCTTTGGCATCTTCTT		
*IL-10*	F:CACTGCTCTATTGCCTGATCTTCC	136	NM_214041.1
	R:AAACTCTTCACTGGGCCGAAG		

^a^*GAPDH*, glyceraldehyde-3-phosphate dehydrogenase; *ZO-1*, zonula occludens-1; *TNF-*α, tumor necrosis factor α; *IL-1*β, interleukin 1β; *IL-2*, interleukin 2; *IL-6*, interleukin 6; *IL-10*, interleukin 10.

**Table 3:** RT-qPCR reaction system and procedures.

Reagent	Volume
SYBR green mixture	5 μL
Forward primers	0.3 μL
Reverse primers	0.3 μL
Nuclease-free water	2.4 μL
cDNA	2 μL

**Table 4:** RT-qPCR reaction procedures.

Reaction stage	Number of cycles	Reaction temperature	Reaction time
Pre-degeneration	1	95 °C	30 s
Cyclic reaction	40	95 °C	5 s
		60 °C	30 s
		72 °C	30 s
Dissolution curve	1	Machine default	Machine default

### Statistical analysis

All experimental data were subjected to one-way analysis of variance (ANOVA) using SPSS 21.0 software (IBM Company, New York, USA). Multiple comparisons among treatments were performed by Tukey's test. *P* < 0.05 was defined as statistically significant difference.

## Results

### Effects of fermented pine needles on growth performance and diarrhea rate of weaned piglets

Table 5 illustrates the effect of dietary fermented pine needles supplementation on growth performance and diarrhea incidence of weaned piglets. No remarkable differences were found in FBW and ADFI on days 1–14, as well as ADFI, ADG, and F/G during days 15–28. The overall ADFI across the whole trial period (days 1–28) also showed no statistical distinction among all treatment groups (*P* > 0.05). Throughout the experiment, piglets in FR1, FR2, and FR3 groups exhibited significantly lower diarrhea rates than the CON group (*P* < 0.05). Both NF and fermented pine needle addition groups (FR1, FR2, FR3) presented significantly higher ADG and better F/G in the early phase (days 1–14) and the whole experimental period (days 1–28) compared with the CON group (*P* < 0.05). Meanwhile, ADG and F/G showed no significant statistical differences among NF, FR1, FR2, and FR3 treatment groups (*P* > 0.05).

**Table 5:** Effects of dietary fermented pine needles supplementation on growth performance and diarrhea rate of weaned piglets.

Items^e^	Treatments^c^					SEM^d^	*P*-value
	CON	NF	FR1	FR2	FR3		
Initial weight, kg	6.25	6.28	6.27	6.25	6.25	0.11	0.92
d 1–14							
FBW (kg)	10.99	11.67	11.85	11.94	11.54	0.15	0.086
ADFI (g/d)	558.69	570.27	570.51	556.37	562.87	8.06	0.315
ADG (g/d)	338.62^b^	385.32^a^	398.96^a^	406.11^a^	399.22^a^	8.35	0.024
F/G	1.65^a^	1.48^b^	1.43^b^	1.37^b^	1.41^b^	0.05	0.006
Diarrhea rate	30.15^a^	12.39^b^	10.60^b^	10.04^b^	10.27^b^	2.09	0.039
d 15–28							
FBW (kg)	18.93^b^	20.07^a^	20.48^a^	20.74^a^	20.12^a^	0.21	0.032
ADFI (g/d)	930.11	942.08	949.29	955.09	943.19	12.06	0.596
ADG (g/d)	567.14	600.05	616.42	628.35	612.86	9.98	0.319
F/G	1.64	1.57	1.54	1.52	1.53	0.05	0.41
Diarrhea rate	15.21^a^	6.42^b^	5.14^b^	4.85^b^	4.92^b^	5.73	0.040
d 1–28							
ADFI (g/d)	744.41	756.18	759.91	755.73	753.03	8.16	0.237
ADG (g/d)	452.88^b^	492.69^a^	507.69^a^	517.23^a^	506.04^a^	7.19	0.033
F/G	1.64^a^	1.53^b^	1.50^b^	1.46^b^	1.49^b^	0.04	0.024
Diarrhea rate	15.08^a^	6.09^b^	5.22^b^	4.99^b^	5.63^b^	0.98	0.029

^ab^Means within the same row that do not share a common superscript are significantly different (*P* < 0.05); *n* = 8.

^c^Control, Control group (basal diet); NF, basal diet with 2% unfermented pine needles; FR1, FR2, and FR3 groups, basal diet with 1.0, 2.0, and 3.0% *Aspergillus niger*-fermented pine needles, respectively.

^d^Standard error of the mean based on pooled estimate of variation.

^e^FBW, Finial body weight; ADFI, Average daily feed intake; ADG, Average daily gain; F/G, Average daily feed intake/Average daily gain.

### Effects of fermented pine needles on the serum D-lactic acid and DAO activity of weaned piglets

Table 6 presents the serum D-lactic acid and DAO concentrations of weaned piglets. On days 14 and 28 of the trial, piglets in FR1, FR2, and FR3 groups exhibited distinctly reduced serum D-lactic acid and DAO levels relative to the CON group (*P* < 0.05). At both sampling time points, no significant differences in these two indicators were observed between the CON and NF groups, except DAO activity on day 28 (*P* > 0.05). In addition, serum D-lactic acid and DAO showed no statistical divergence between all FR treatments and the NF group (*P* > 0.05).

**Table 6:** Effects of dietary fermented pine needles supplementation on serum D-lactic acid and DAO level of weaned piglets.

Items	Treatments^c^					SEM^d^	*P*-value
	CON	NF	FR1	FR2	FR3		
d 14							
D-lactic acid (μg/mL)	1.36^a^	1.18^ab^	1.09^b^	1.07^b^	1.21^ab^	0.13	0.040
DAO (pg/mL)	269.38^a^	243.23^ab^	190.67^b^	186.94^b^	225.86^ab^	11.29	0.034
d 28							
D-lactic acid (μg/mL)	1.55^a^	1.36^ab^	1.24^b^	1.20^b^	1.30^ab^	0.10	0.039
DAO (pg/mL)	446.38^a^	349.64^b^	280.31^ab^	260.09^c^	328.49^b^	16.38	0.042

^ab^Means within the same row that do not share a common superscript are significantly different (*P* < 0.05); *n* = 8.

^c^Control, Control group (basal diet); NF, basal diet with 2% unfermented pine needles; FR1, FR2, and FR3 groups, basal diet with 1.0, 2.0, and 3.0% *Aspergillus niger*-fermented pine needles, respectively.

^d^Standard error of the mean based on pooled estimate of variation.

### Effects of fermented pine needles on the iNOS, TNOS, and NO concentrations of weaned piglets

Table 7 presents the levels of iNOS, TNOS, and NO in serum and jejunal mucosa of weaned piglets. Serum NO concentrations were significantly lower in the FR1, FR2, and FR3 groups than in the CON and NF groups on days 14 and 28 of the experiment (*P* < 0.05). At day 28 in serum and day 14 in jejunal mucosa, TNOS activity declined significantly in the three fermented pine needle groups when compared with the CON and NF groups (*P* < 0.05). On days 14 and 28, no obvious differences in serum and jejunal mucosal iNOS levels were found among all fermented treatment groups (*P* > 0.05). No statistical variations were observed in serum TNOS activity at day 14 and jejunal mucosal TNOS activity at day 28 across all experimental groups (*P* > 0.05). Additionally, jejunal mucosal NO contents at both time points showed no notable divergence among all treatments (*P* > 0.05).

**Table 7:** Effects of fermented pine needles on iNOS, TNOS, and NO concentrations in serum and jejunal mucosa of weaned piglets.

Period	Items^e^		Treatments^c^					SEM^d^	*P*-value
			CON	NF	FR1	FR2	FR3		
D 14	Serum(U/mL)	iNOS^e^	15.86	15.79	15.62	15.50	15.56	1.56	0.136
		TNOS^e^	20.05	19.94	18.87	18.19	18.64	2.03	0.054
		NO^e^	58.16^a^	35.62^b^	30.45^b^	28.46^b^	29.97^b^	5.96	0.031
	Jejunal mucosa (U/mg prot)	iNOS^e^	0.64	0.58	0.53	0.50	0.51	0.03	0.103
		TNOS^e^	1.86^a^	1.67^ab^	1.56^b^	1.42^b^	1.50^b^	0.20	0.024
		NO^e^	3.36	3.21	3.16	3.09	3.14	0.92	0.421
D 28	Serum (U/mL)	iNOS^e^	16.01	15.74	15.53	15.02	15.38	2.94	0.267
		TNOS^e^	29.58^a^	20.97^ab^	18.39^b^	18.06^b^	18.62^b^	2.39	0.041
		NO^e^	96.45^a^	89.92^ab^	77.69^b^	75.34^b^	78.60^b^	9.38	0.028
	Jejunal mucosa (U/mg prot)	iNOS^e^	0.58	0.49	0.42	0.38	0.40	0.05	0.084
		TNOS^e^	1.67	1.52	1.49	1.47	1.58	0.06	0.071
		NO^e^	3.18	3.01	2.86	2.64	2.91	0.37	0.069

^ab^Means within the same row that do not share a common superscript are significantly different (*P* < 0.05); *n* = 8.

^c^Control, Control group (basal diet); NF, basal diet with 2% unfermented pine needles; FR1, FR2, and FR3 groups, basal diet with 1.0, 2.0, and 3.0% *Aspergillus niger*-fermented pine needles, respectively.

^d^Standard error of the mean based on pooled estimate of variation.

^e^iNOS, inducible nitric oxide synthase; TNOS, total nitric oxide synthase; NO, nitric oxide.

### Effects of fermented pine needles on the cytokines concentration in serum and jejunal mucosa of weaned piglets

Table 8 illustrates the impacts of fermented pine needles on serum and jejunal mucosa cytokine contents of weaned piglets. On trial day 14, the FR1, FR2, and FR3 groups had significantly lower serum and jejunal mucosal levels of TNF-α, IL-1β, IL-2, and IL-1β compared with the CON group, while jejunal mucosal IL-1β showed no significant difference (*P* < 0.05). At this time point, serum and jejunal mucosal TNF-α, IL-1β, and IL-2 exhibited no measurable differences between the CON and NF groups (*P* > 0.05), and these inflammatory indicators also remained similar between NF and each FR treatment (*P* > 0.05).

**Table 8:** Effects of fermented pine needles on the cytokines concentration in serum and jejunal mucosa of weaned piglets.

Period	Items		Treatments^c^					SEM^d^	*P*-value
			CON	NF	FR1	FR2	FR3		
D 14	Serum (pg/mL)	*TNF-α*	28.69^a^	20.38^ab^	16.16^b^	15.38^b^	15.62^b^	1.92	0.024
		*IL-1β*	0.58^a^	0.52^ab^	0.49^b^	0.45^b^	0.48^b^	0.08	0.021
		*IL-2*	32.98^a^	27.32^ab^	22.45^b^	20.94^b^	21.51^b^	3.68	0.038
		*IL-6*	128.67^a^	100.35^ab^	97.65^b^	83.34^b^	98.34^b^	10.23	0.024
		*IL-10*	31.68^b^	39.57^ab^	43.68^a^	46.95^a^	45.87^a^	2.37	0.039
	Jejunal mucosa (U/mg prot)	*TNF-α*	38.51^a^	34.04^ab^	32.61^b^	31.19^b^	32.47^b^	0.42	0.016
		*IL-1β*	114.39	110.16	105.67	102.31	104.68	3.38	0.367
		*IL-2*	356.37^a^	312.05^ab^	295.34^b^	268.57^b^	284.19^b^	25.37	0.040
		*IL-6*	124.39^a^	110.61^ab^	108.38^b^	101.24^b^	106.39^b^	3.04	0.238
		*IL-10*	100.35^b^	125.37^ab^	139.67^a^	150.14^a^	140.27^a^	10.38	0.031
D 28	Serum (pg/mL)	*TNF-α*	46.92	45.83	45.32	45.03	45.01	2.94	0.225
		*IL-1β*	0.67^a^	0.61^ab^	0.53^b^	0.46^b^	0.51^b^	0.03	0.036
		*IL-2*	29.67	29.64	28.67	29.12	29.71	1.85	0.902
		*IL-6*	105.38^a^	93.12^b^	83.41^b^	74.93^b^	80.34^b^	2.94	0.035
		*IL-10*	35.63b	43.67^ab^	48.82^a^	50.37^a^	50.34^a^	1.75	0.023
	Jejunal mucosa (U/mg prot)	*TNF-α*	52.46^a^	44.28^ab^	40.38^b^	38.31^b^	40.49^b^	0.27	0.024
		*IL-1β*	134.24	116.24	113.34	112.19	112.18	4.37	0.306
		*IL-2*	386.48^a^	321.32^ab^	300.27^b^	288.96^b^	300.21^b^	28.62	0.035
		*IL-6*	162.18^a^	151.18^ab^	145.27^b^	140.61^b^	142.45^b^	4.74	0.351
		*IL-10*	140.42^b^	175.32^ab^	189.74^a^	200.01^a^	187.39^a^	12.78	0.012

^ab^Means within the same row that do not share a common superscript are significantly different (*P* < 0.05); *n* = 8.

^c^Control, Control group (basal diet); NF, basal diet with 2% unfermented pine needles; FR1, FR2, and FR3 groups, basal diet with 1.0, 2.0, and 3.0% *Aspergillus niger*-fermented pine needles, respectively.

^d^Standard error of the mean based on pooled estimate of variation.

On day 28, the three fermented pine needle groups presented significantly decreased serum IL-1β and jejunal mucosal TNF-α levels relative to the CON group (*P* < 0.05). By comparison, day 28 serum TNF-α and jejunal mucosal IL-1β did not differ statistically among CON, NF and FR treatments (*P* > 0.05). At both detection time points, FR1, FR2, and FR3 groups had significantly higher serum and jejunal mucosal IL-10 contents than the CON group (*P* < 0.05). Serum and jejunal mucosal IL-6 concentrations were significantly reduced in fermented groups vs. CON at day 14 and day 28 (*P* < 0.05). For serum and jejunal mucosal IL-6 and IL-10 (excluding serum IL-6 on day 28), no obvious differences existed between CON and NF groups on days 14 and 28 (*P* > 0.05); likewise, the NF group showed no statistical distinction from any FR treatment for these indexes (*P* > 0.05).

### Effects of fermented pine needles on the mRNA expression of the tight junction protein in jejunal mucosa of weaned piglets

**Figure 1:**
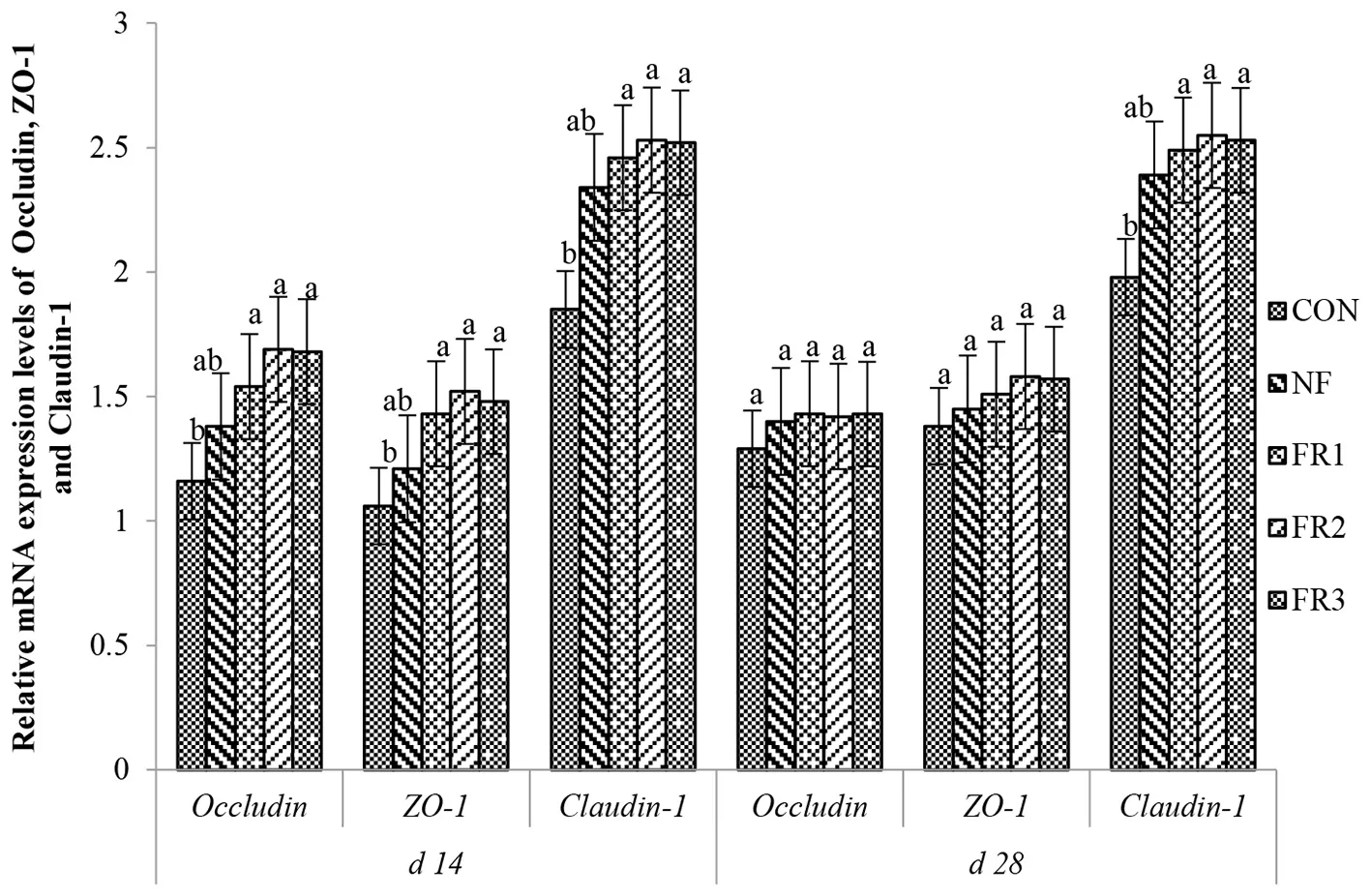
Effects of fermented pine needles on the relative mRNA expression levels of the tight junction proteins in the jejunal mucosa of weaned piglets. ^ab^Means within the same row that do not share a common superscript are significantly different (*P* < 0.05); *n* = 8. Control, Control group (basal diet); NF, basal diet with 2% unfermented pine needles; FR1, FR2, and FR3 groups, basal diet with 1.0, 2.0, and 3.0% *Aspergillus niger*-fermented pine needles, respectively. Standard error of the mean based on pooled estimate of variation.

Figure 1 displays the influences of fermented pine needles on the mRNA expression of tight junction proteins in the jejunal mucosa of weaned piglets. On days 14 and 28, jejunal mucosal *Occludin, ZO-1* and *Claudin-1* mRNA expression were upregulated in FR1, FR2, and FR3 vs. CON (*P* < 0.05), and *Claudin-1* only differed significantly on day 28. On day 14, the three genes showed similar expression between CON and NF, as well as between NF and FR treatments (*P* > 0.05). On day 28, *Occludin* and *ZO-1* expression had no obvious difference between CON and NF, and no divergence was found between NF and FR groups either (*P* > 0.05).

### Effects of fermented pine needles on the mRNA expression of inflammatory cytokines related genes in jejunal mucosa of weaned piglets

**Figure 2:**
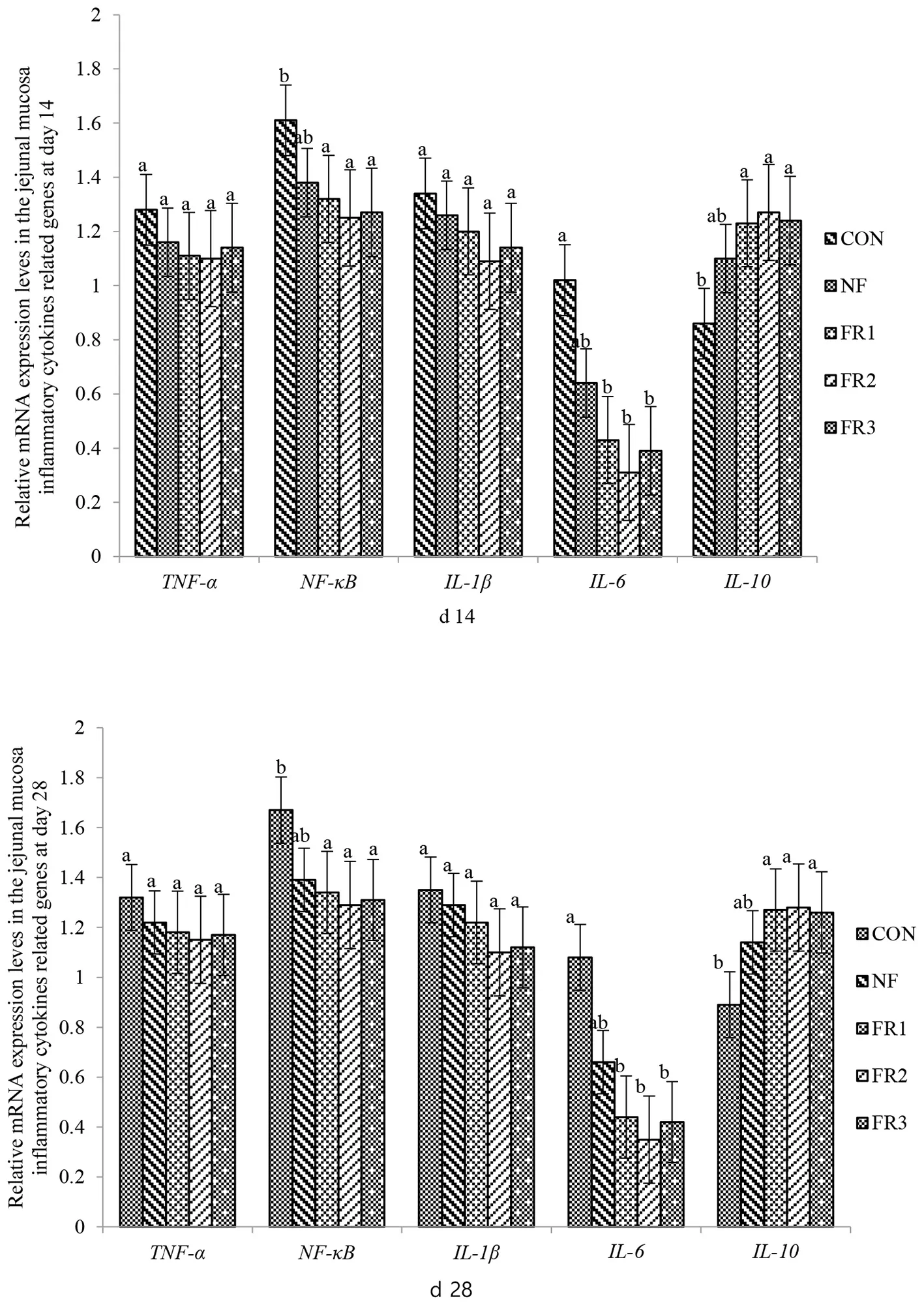
Effects of fermented pine needles on the mRNA expression of inflammatory cytokines related genes in jejunal mucosa of weaned piglets. ^ab^Means within the same row that do not share a common superscript are significantly different (*P* < 0.05); *n* = 8. Control, Control group (basal diet); NF, basal diet with 2% unfermented pine needles; FR1, FR2, and FR3 groups, basal diet with 1.0, 2.0, and 3.0% *Aspergillus niger*-fermented pine needles, respectively. Standard error of the mean based on pooled estimate of variation.

Figure 2 illustrates the impacts of fermented pine needles on the mRNA expression of related genes in the jejunal mucosa of weaned piglets. Relative to the CON group, jejunal mucosal *IL-10* mRNA expression was significantly upregulated in FR1, FR2, and FR3 at days 14 and 28 (*P* < 0.05). By contrast, the transcript levels of *NF-*κ*B* and *IL-6* were obviously suppressed in the three fermented pine needle groups at both time points (*P* < 0.05). There were no significant differences in the expression of *NF-*κ*B, IL-10*, and *IL-6* were observed between the CON and NF groups on the two detection days (*P* > 0.05). Similarly, the expression levels of these genes remained comparable between NF and each FR treatment (*P* > 0.05).

## Discussion

Growth performances and diarrhea incidence are core production indicators for weaned pig breeding. Previous studies have confirmed that dietary pine needles supplementation improves growth performance of quail (18) and laying hens (13, 19). In the present study, both raw and fermented pine needle supplementation significantly increase ADG and reduced F:G of weaned piglets during days 1–14 and 1–28, and effectively alleviate weaning diarrhea. Consistent with previous reports in poultry, these beneficial effects are likely attributed to abundant bioactive substances in pine needles including monosaccharides, organic acids, terpenoids and flavonoids, which exert antibacterial, antioxidant and immune-regulating functions (13, 15, 20). Moreover, our previous research verified that *Aspergillus niger* fermentation produces extracellular enzymes to degrade condensed tannins in pine needles, promoting feed digestion and absorption and relieving cellular oxidative stress (15).

Intestinal mucosal barrier integrity is the core defensive line of animal intestinal health. Once mucosal structure is damaged, intestinal immune tissue will be directly exposed to intestinal luminal antigens and trigger inflammatory responses (21). Serum DAO and D-lactic acid are common detection indicators to reflect intestinal mucosal damage degree, but neither marker can independently represent specific intestinal permeability changes. Previous stress trials in broilers reported elevated serum D-lactic acid and DAO after intestinal injury, while intestinal protective feed additives can reduce the concentration of these two indicators (22–24). Consistent with existing literature, both raw and fermented pine needles supplementation reduced serum D-lactic acid and DAO levels in this trial, indicating pine needle materials can maintain normal intestinal mucosal structure and reduce mucosal damage, which is mainly related to polyphenol active substances in pine needles. Numerous studies have confirmed that polyphenol-enriched natural materials, including green tea, cocoa and various fruits, can strengthen intestinal barrier stability (25). Pine needles used in this experiment are rich in bioactive polyphenols such as flavonoids and anthocyanins, compounds proven to improve intestinal barrier function (15). Accordingly, these functional ingredients may be primarily responsible for the protective effects on mucosal integrity observed in the current work. Such conclusions are further supported by the altered expression profiles of intestinal tight junction proteins detected in this study.

Tight junction protein are key molecules maintaining intestinal barrier homeostasis. Our results showed that both raw and fermented pine needles significantly upregulated the transcriptional levels of jejunal mucosal ZO-1, Claudin-1 and Occludin. Higher abundance of tight junction proteins can maintain epithelial barrier stability and reduce intestinal permeability. Existing research have confirmed that polyphenolic substances can positively regulate tight junction protein gene expression, indicating that additives rich in polyphenols can effectively enhance the gene expression of Claudin and ZO-1 (25, 26). The favorable changes in tight junction gene expression observed in this study are likely linked to the intrinsic polyphenol components in pine needles and their fermented products, which exert protective effects on intestinal mucosal barrier and present good application potential for alleviating intestinal injury in weaned piglets. An important limitation should be clarified here: mRNA expression of tight junction proteins has limited correlation with final protein abundance and physiological function, since these proteins undergo extensive post-translational modification and redistribution under weaning stress. Further research will detect protein expression and cellular localization to supplement this mechanism. Even so, the precise molecular pathways through which raw and fermented pine needles regulate the expression and physiological activity of ZO-1, claudin-1 and occludin are still not fully understood. It is necessary to carry out further in-depth experiments to clarify the regulatory effects of pine needle derivatives on tight junction proteins. Subsequent molecular studies should focus on exploring how fermented pine needles alter the distribution of tight junction proteins, and further mediate the migration and physiological function of intestinal epithelial cells.

Intestinal tight junction protein expression is closely linked to pro-inflammatory cytokines secretion. The current results showed that dietary raw and fermented pine needles significantly lowered serum and jejunal mucosal concentrations of pro-inflammatory TNF-α, IL-1β, and IL-2, while significantly elevating anti-inflammatory IL-10 levels and reducing IL-6 contents in serum and jejunal mucosa. The above trend is consistent with Wang et al. (11) and Guo et al. (13), who reported pine needle extracts improve immune status of poultry by regulating inflammatory cytokines. Overall, pine needle intervention inhibited the production and release of proinflammatory TNF-α and promoted IL-10 secretion, thus alleviating intestinal inflammatory damage; this regulatory trend matched the increased expression of intestinal tight junction proteins. Previous research has confirmed that fermented pine needles are rich in terpenoids, lipids, saccharides and essential oils, all of which exert strong antioxidant and immune regulatory activities (27, 28). In the present experiment, groups fed fermented pine needles had lower proinflammatory cytokine levels (TNF-α, IL-1β, IL-2) and higher IL-10 abundance than the unfermented group (11). These favorable effects are probably attributable to abundant bioactive substances produced during *Aspergillus niger* fermentation, which further enhance the antioxidant capacity and immune modulation ability of fermented pine needle products. Dietary fermented pine needles elevated jejunal *IL-10* gene expression and simultaneously suppressed the transcription of *NF-*κ*B* and *IL-6*. Such gene expression changes were consistent with the variation trend of cytokines in serum and jejunal tissue, showing decreased TNF-α, IL-1β and IL-2 along with increased IL-10. Furthermore, the activated NF-κB can initiate a positive feedback pathway, thereby promoting the production of TNF-α. This molecule further drives the synthesis and release of downstream proinflammatory factors, eventually impairing the structural integrity of the jejunal mucosal barrier. In this study, fermented pine needle supplementation reduced both protein content and gene expression of proinflammatory cytokines, helping relieve excessive inflammatory response in weaned piglets. Meanwhile, fermented pine needles may also suppress NF-κB activity by regulating the level and transcription of IL-10 and TNF-α. The above findings suggested that fermented pine needles could favorably modulate the immune status of weaned piglets. The regulatory effects on inflammatory cytokine contents and their gene expression in piglets fed fermented pine needles may be ascribed to abundant essential oils, terpenoids and polyphenols contained in pine needle fermented products (11).

Some existing studies have pointed out that pine needles can enhance growth performance and regulate serum biochemical indices including IL-6, IL-1β, and TNF-α. Its inhibitory effect on inflammatory factors is closely related to the enrichment of beneficial intestinal microbes such as *Butyricicoccus, Barnesiella*, and *Oscillibacter*, as well as the suppression of harmful strains like *Fusobacterium* and *Anaerobiospirillum* (13, 20, 29). At present, relevant studies regarding the regulatory role of pine needles, especially fermented products, on the intestinal microflora of weaned piglets remain insufficient. Future research should further explore the antibacterial potential of fermented pine needles, as well as their modulatory action on the proliferation of beneficial intestinal flora in weaned piglets.

Nitric oxide serves as a key signaling molecule regulating immune reaction and *in vivo* oxidation balance (15, 30).Polyphenol substances can inhibit NF-κB binding to the iNOS promoter, reduce iNOS transcription and NO production, and relieve inflammatory stress (15, 31, 32). In this trial, fermented pine needles had no significant influence on serum and jejunal mucosal iNOS levels, but significantly reduced serum NO concentration and tissue TNOS activity compared with CON and NF groups, indicating fermented pine needles alleviate oxidative stress by inhibiting NO and TNOS. Pine-derived proanthocyanidins can scavenge NO free radicals, which provides a reasonable theoretical basis for the antioxidant effects observed in the current trial (33). At present, research focusing on fermented pine needles regulating weaned piglet intestinal microflora is still insufficient. Follow-up trials will further explore the effects of fermented pine needles on intestinal beneficial flora proliferation and antibacterial activity.

## Conclusion

Overall, dietary supplementation with pine needles (raw or fermented) improves growth performance and reduces diarrhea incidence in weaned piglets. As natural feed additive, pine needles positively regulate antioxidant capacity, immune function and intestinal mucosal barrier integrity of piglets via modulating nitric oxide synthase activity, optimizing jejunal barrier structure, and balancing intestinal cytokines secretion and related related functional gene expression. Fermented pine needles exhibit equivalent beneficial effects compared with raw pine needles under the present experimental conditions, and fermentation treatment does not generate extra significant improvement effect. Fermented pine needles show promising application potential as green feed supplements for weaned pig production.

## Data Availability

The original contributions presented in the study are included in the article/supplementary material, further inquiries can be directed to the corresponding author.
